# Cerebral lesions in the central pain matrix are associated with headache in multiple sclerosis

**DOI:** 10.1038/s41598-025-93869-7

**Published:** 2025-03-23

**Authors:** Kilian Fröhlich, Kosmas Macha, Gabriela Siedler, Alexander Sekita, David Haupenthal, Anne Mrochen, Ruihao Wang, Leah Schembs, Arnd Dörfler, Frank Seifert, Stefan Schwab, Klemens Winder

**Affiliations:** 1 Departments of Neurology, University Hospital Erlangen, Schwabachanlage 6, 91054 Erlangen, Germany; 2 Bellevue Medical Group, Theaterstrasse 8, 8001 Zürich, Switzerland; 3 Departments of Neuroradiology, University Hospital Erlangen, Schwabachanlage 6, 91054 Erlangen, Germany

**Keywords:** Headache, Multiple sclerosis, Cerebral lesions, Voxel-based lesion symptom mapping, Imaging, Magnetic resonance imaging, Neuroscience, Neurology

## Abstract

Headache is very frequent in multiple sclerosis. However, the question whether headache is just coincidental or may be secondary due to inflammatory cerebral multiple sclerosis lesions is yet to be clarified. This study intended to evaluate the distribution of cerebral lesion sites and the potential presence of specific lesion clusters in patients with multiple sclerosis and comorbid headache using voxel-based lesion symptom mapping (VLSM). Patients with multiple sclerosis and headache were prospectively identified and included in a university neurological center between 2017 and 2023. Only patients with headache onset after first manifestation of multiple sclerosis were included. Demographic and clinical data were assessed, and lesion volumes calculated. Cerebral lesion sites were correlated voxel-wise with presence and absence of headache using non-parametric permutation testing. A cohort of multiple sclerosis patients served as controls for the VLSM-analysis. 48 multiple sclerosis patients with headache were included, as well as 92 controls without headache. Of the 48 patients with headache, 39 (81%) were female and nine (19%) were male. Mean age was significantly higher in headache patients than in controls (51 + / − 11 vs. 42 + / − 11 years, p < 0.05). EDSS, disease duration and lesion volumes did not significantly differ between both groups. Lesion overlap of all patients demonstrated a distribution of white matter lesions consistently in all subcortical brain areas. The VLSM-analysis showed associations between headache and lesion clusters in the left insula, left hippocampus and right thalamus. In our study, multiple sclerosis lesions in the left insula, left hippocampus and right thalamus were associated with headache in multiple sclerosis patients. The data therefore indicates that headache in multiple sclerosis may, in a proportion of patients, result from lesions in the central nervous systems’ pain processing network.

**Trial registration**: No. 93_17 B, Ethics committee of the University Hospital Erlangen-Nürnberg.

## Background

Multiple Sclerosis (MS) is an autoimmune inflammatory disease of the central nervous system which pathogenesis is not completely understood^1^. Headache prevalence is significantly higher in MS patients^2,3^. For example, migraine is reported more than twice in MS patients compared to controls^4^.

Both conditions severely affect the quality of life of affected patients, yet the background of their association has not been fully elucidated^4–6^.

In some cases, headache in MS may be coincidental or may represent a side-effect of disease-modifying drugs in MS^5,7^. However, previous studies suggested a causal relationship between headache and MS, i.e. that headache might also be secondary and present a symptom in MS, resulting from inflammatory MS activity in the CNS^2,8^.

Migraines have been associated with subcortical white matter lesions supra- and infratentorially before^9–15^. Some studies have demonstrated a two- to fourfold prevalence of white matter lesions in patients with migraine compared to controls^16^. As neuroinflammation plays an important role in both migraine and MS, inflammatory MS activity may facilitate the development of headache^16,17^.

Previous data indicates that in certain cases, strategic MS lesions in headache-relevant cerebral pathways may initiate headache^16^. In particular, migraine onset has been observed to be associated with lesion formation in the trigeminal root entry zone and periaqueductal gray matter^9,10,12^. The trigeminocervical complex is composed of major relay neurons for nociceptive afferent input from the meninges and cervical structures that are important for headache and the periaqueductal gray is an important structure for pain modulation^12,14^. MS patients with lesions in the periaqueductal gray matter have been shown to display a four-fold increase in migraine-like headaches^9^.

In other, e.g. non-inflammatory cerebral diseases, parenchymal lesions are already known to lead to symptomatic headache and are recognized as important clinical symptoms. In ischemic stroke, for example posterior lesions have shown to be associated with headache^18^, possibly due to strategic lesions in the central pain matrix.

Although providing important insights, previous works in MS patients consist only of case series or a visual region-of-interest-based descriptive analysis with a focus on infratentorial brain regions^12,13^. Systematic data and especially neuroimaging evidence about the association between inflammatory MS lesions and symptomatic headache in MS is still scarce^17,19,20^.

To overcome the mentioned disadvantages of previous studies, we therefore performed a statistical voxel-based lesion mapping analysis of the whole brain to identify brain regions which are related to the development of headache in multiple sclerosis^21^. Statistical imaging analysis like the voxel-wise lesion symptom mapping (VLSM) allows investigating voxel-by-voxel associations between cerebral lesion location and an outcome without having any a priori hypothesis^16,21–23^.

We hypothesized that there are specific lesion patterns, which are associated with the development of headache in multiple sclerosis.

## Methods

The aim of this lesion mapping study was to identify brain regions, which are related to the development of headache in multiple sclerosis.

### Patients

The prospective study was approved by the local ethics committee of the Friedrich-Alexander University Erlangen-Nuremberg (No. 93_17 B). All procedures were carried out in accordance with the relevant guidelines and regulations of our university ethics committee and the Declaration of Helsinki. Informed consent was obtained from all subjects or their legal guardian.

For the study, MS patients admitted to the Department of Neurology at the University Hospital Erlangen were prospectively screened for headache between 2017 and 2023 and included, if suitable.

To exclude patients with coincidental comorbid presence of headache and MS to the maximum possible extent, only patients with headache onset after the first manifestation of MS were included. Patients who had a history of other cerebral conditions or whose headache was interpreted as a side effect of the treatment with disease-modifying MS drugs were excluded. Patients with analgesic rebound headache were also excluded. Only patients with persisting headache at inclusion were considered.

The diagnosis of MS followed the recent guidelines^1,24^. Disability was rated with the EDSS scale^25^.

Headache characteristics were evaluated via a written questionnaire adhering to the International Classification of Headache Disorders, which was used for the diagnosis of the headache type^26^. Headache patients were subclassified into three groups (migraine, tension headache, others). Detailed medical history of the patients, including medication, secondary diagnoses, laboratory results and radiological examinations was assessed. Vital signs were obtained and an ECG was performed.

As a control group, a cohort of MS patients without headache was established. All data were entered in a prospective database.

### Cerebral imaging

All patients underwent magnetic resonance imaging (MRI) (3 Tesla, Magnetom Trio or 1.5 Tesla Siemens Magnetom Sonata, Siemens Healthcare, Erlangen, Germany) of the brain.

### VLSM

Two experienced investigators (K.F. and KW.) delineated the boundaries of the hyperintense flair lesions on anonymized imaging scans using MRIcron (www. mrico.com)^23^. Both raters were blinded to clinical parameters during imaging analysis. The MRI scan and the lesion shape were transferred into stereotaxic space using the normalization algorithm of SPM12 (http://www.fil.ion.ucl.ac.uk/spm/) and the Clinical Toolbox for SPM (http://www.mricro.com/clinical-toolbox/spm8-scripts). Using the MR-segment-normalize algorithm of the Clinical Toolbox, the MR images were transformed to the T1 template with a resampled voxel size of 1 × 1 × 1 mm^3^ ^27^. Lesion volumes in voxels were calculated using the non-parametric mapping (NPM) algorithm included in MRIcron. In a VLSM analysis, the lesion site was correlated with the occurrence of headache using non-parametric permutation testing^22^. All lesioned voxels were included in the analysis. A false discovery rate (FDR) correction of 0.05 was applied. The peak coordinates of the involved regions are presented in Montreal Neurological Institute (MNI)-space. The version AAL of the automated anatomic labelling atlas was used to determine the localization of the affected brain regions^28^.

### Statistical analysis

For data analysis, a commercially available statistic program (SPSS 20.0; IBM, Armonk, NY) was used. Distribution of data was tested using Shapiro–Wilk test. Data are presented as mean and standard deviation (SD) or median and interquartile range (IQR). Normally distributed patient and control data were compared using the *t*-test for unpaired samples. Non-normally distributed data were compared using the Mann–Whitney *U*-test. Significance was assumed for P < 0.05.

## Results

### Patient characteristics

48 of the identified MS patients with headaches agreed to participate in the study and were included. Four patients were excluded because headaches were interpretated as a side effect of the disease modifying MS medication. No patients were excluded because of comorbid other cerebral diseases. 92 MS patients were recruited as controls.

Clinical characteristics of the study participants are described in Table 1. Of the 48 patients with headache, 39 (81%) were female and 9 (19%) were male. Among controls, 66 (72%) were female and 26 (28%) were male.

**Table 1:** Characteristics of the 48 individuals with headache and the 92 controls without headache in patients with multiple sclerosis.

Characteristics	Headache (n = 48)	No headache (n = 92)
Age, mean ± SD; years	51 ± 11*	42 ± 11*
Sex		
M	9	26
F	39	66
EDSS, median (IQR)	3 (1–4)	2 (1.5–4.5)
Disease duration, median (IQR)	61 (15–125)	36 (12–123)
MS type		
PPMS	1	3
RRMS	41	66
SPMS	4	22
Lesion volume (voxels), median (IQR)	20,586 (5735–32,889)	16,787 (9422–37,365)
Migraine	22	
Tension headache	19	
Others	7	

*IQR* interquartile range, *M/F* man/female ratio, *SD* standard deviation.

*Indicates significant difference using Mann Whitney *U* test.

Mean age was significantly higher in headache patients than in controls (51 + / − 11 vs. 42 + / − 11 years, p < 0.05).

Median EDSS score, disease duration and lesion volume were higher in the headache cohort (Table 1), yet these results did not reach statistical significance.

### VLSM

**Fig. 1:**
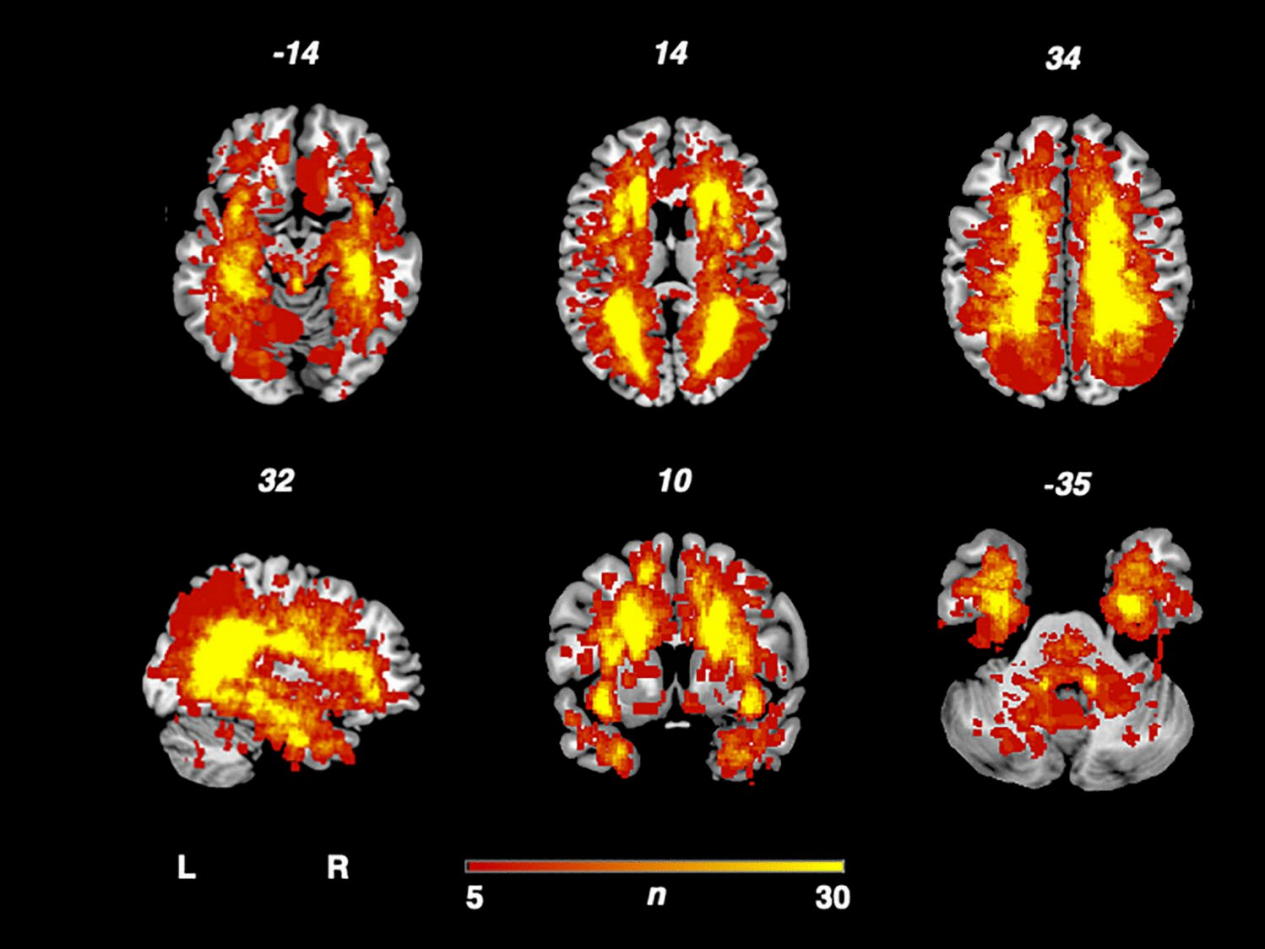
Overlap and distribution of T2 lesions of all patients. The number of overlapping lesions is illustrated by color coding. The pattern shows the highest overlay in subcortical and periventricular regions, congruent with the multiple sclerosis cohort. *L* left, *n* number of individuals with a lesion in each voxel, *R* right.

In Fig. 1, the overlap of cerebral lesions of all 140 MS patients is demonstrated. The analysis derived a typical lesion pattern of inflammatory MS lesions with subcortical preponderance, which is illustrated in axial, sagittal and coronar view.

**Fig. 2:**
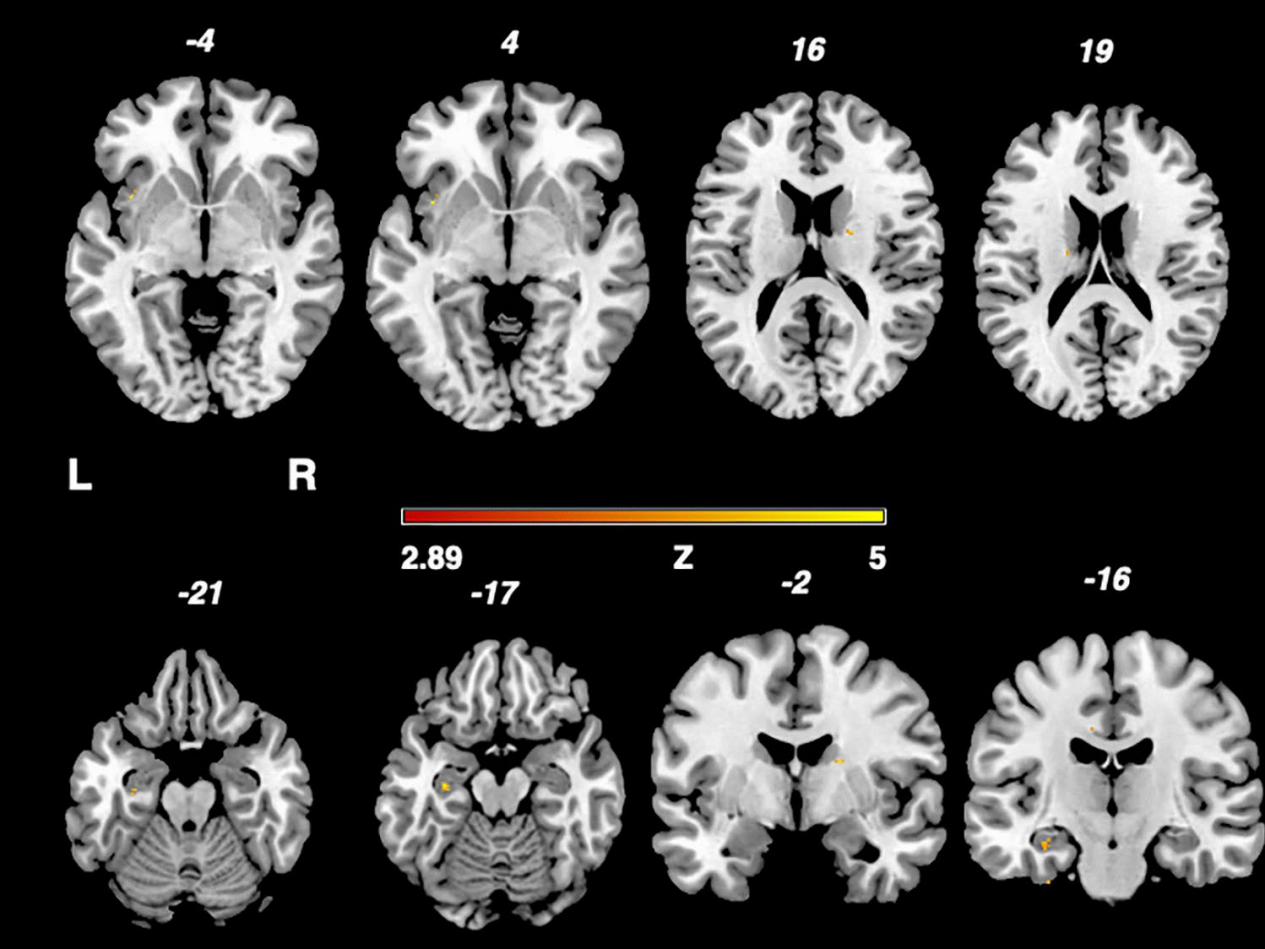
Depicition of the nonparametric Liebermeister test results. Associations of lesioned voxels with headache were found in the left insula, left hippocampus and right thalamus. Only voxels that were damaged in at least two patients were included in the Liebermeister test analysis. A family wise error (FWE) correction of p < 0.05 was applied. *L* left, *z* z-score, *R* right.

Figure 2 shows the results of the nonparametric Liebermeister analysis, producing three lesion clusters associated with brain areas attributed to the central nervous pain network: The left insula, right thalamus and left hippocampal region.

## Discussion

The comorbidity of MS and headache and the question, whether this association is causal or coincidental, has an immense clinical impact and hasn’t been finally answered yet. Previous work suggested that certain, especially brainstem lesions may be associated with headache and therefore headache may be symptomatic in some cases^9,11,12^. Yet, these studies used different methods, but not a statistical and controlled, voxel-wise approach of the whole brain^29^.

Therefore, the aim of this study was to identify lesioned brain regions associated with headache in multiple sclerosis using novel VLSM techniques. To the best of our knowledge, this is the first statistical lesion mapping study on a voxelwise basis of the whole brain investigating headache in multiple sclerosis.

Our study revealed as a major finding lesion cluster in the right thalamus, the left insula and the left hippocampus associated with headache in MS patients (Fig. 2). All these structures are known to be important elements of the central pain network, crucial for the central nervous processing of afferent pain signals. Lesions of those central nervous system structures that are involved in pain processing are known to potentially lead to alterations of pain sensation or centrally mediated pain^21,30–32^.

In short, painful stimuli are conveyed to the periaqueductal gray matter (PAG) in the brainstem and relayed to the thalamus, then being projected to the insula, somatosensory cortex and other brain regions^32^.

First, we detected a lesion cluster in the bilateral thalamus. It has been recognized that lesions of the thalamus can result in post-stroke pain syndromes^30^. Disturbed connectivity patterns between pain processing areas and the pain modulatory system may contribute to the generation of pain states^33^. As the thalamus exerts inhibitory effects via a neural pathway on the insula and the PAG^30^, we propose that a disinhibition of the endogenous pain modulation by localized thalamic lesions may facilitate the development of headache.

Second, the analysis yielded lesions in the left insula (Fig. 2).

The insula is crucial for integrating and encoding nociceptive signals^31,32,34,35^. While the posterior insula contributes to encoding pain intensity, the anterior insular cortex seems to be particularly involved in the emotional processing of pain^32,34,36–38^. Furthermore, the anterior insular cortex is involved in the cortical control of endogenous pain modulatory systems^33,34^. A key brain region of the descending pain system is the PAG. The PAG receives input from anterior insular cortex and is known to exert antinociceptive effects on the perception of noxious stimuli^32,39,40^.

In our study, we found an association between the occurrence of headache in MS patients and lesions predominantly in the anterior insular cortex. Therefore, an altered connectivity profile of these brain areas due to insular MS lesions may increase susceptibility to painful signals.

Third, we found that lesions in the left hippocampus are associated with headache in MS patients (Fig. 2). The hippocampus is part of the central pain matrix and linked to other pain processing regions via pathways, namely the insula^37,40^. It plays an important role in pain inhibition and modulation, especially involving the affective system and cognition^41–43^. The study by Ploghaus et al.^44^ indicates that, during states of heightened anxiety, the hippocampus can amplify aversive sensation.

Especially the left hippocampus, as affected in our study, is responsible for the development of hyperalgesia and the emergence of persistent pain^42,43^. Hence, we hypothesize that lesions in the hippocampus may promote headache by disinhibition of central inhibitory pain mechanisms^32^.

MS patients with headaches were older than the controls (Table 1). Additionally, although beyond the level of significance, our study showed a tendency towards a higher lesion load, EDSS and disease duration among headache patients. This may be indicators that MS patients with headache may have a higher inflammatory activity and a more severe MS course, which has been discussed before^45,46^. However, even in previous studies, where patients with headache reported more MS or comorbid symptoms, this did not translate into a higher MS disability scale or a different lesion load^12,46^. The authors postulated that this fact could be best explained by an altered pain perception and decreased pain threshold due to central sensitization in the headache group^46^.

In our opinion, impaired pain processing would be well explained by the circumscribed lesion clusters in distinct brain regions of the central pain network like the insula, thalamus and hippocampus, as we found in our study.

Previous brought evidence that in distinct cases, brainstem lesions are associated with headache in MS^9,12,13^. Interestingly, these findings were not confirmed in our study. However, these case studies/series and a study via visual ROI-analysis suggest that migraine in MS may result from MS brainstem lesions in some distinct cases, but do only partially explain the increased prevalence of migraine in MS^9,13^. As VLSM is a very robust method, needing enough lesion overlap coverage to produce significant association in the Liebermeister test. Infratentorial and brainstem lesion load, as depicted in Fig. 1, is lower than in supratentorial and periventricular lesions. Based on our results we therefore think that headache in MS may be associated with brainstem lesions in distinct, yet not in frequent patients.

Taken together, our study provides evidence of an association of lesions in the central nociception pathways with headaches in MS. However, in everyday clinical practice, it remains hard to determine wether headaches are primary and comorbid or secondary in MS – a subject that needs to be addressed in further studies^7^.

### Limitations

Although reported headaches were an exclusion criterion, we cannot completely exclude that headaches were also present in the control group. Due to the heterogeneity of headache and the fact that diagnosis is based on clinical features, we also cannot exclude that in a small proportion of patients, headaches may be due to the disease modifying MS medication in the study cohort, although this was an important exclusion criterion. Unfortunately, including covariates, the Liebermeister test yielded no significant results, most likely owing to low statistical power. Additionally, the cohort was too small for subgroup analyses. A larger study cohort may have produced more significant results.

## Conclusions

In our study, MS lesions in the left insula, left hippocampus and bilateral thalamus were associated with headache in MS patients. The data therefore indicates that headache in MS may, in a proportion of patients, result from lesions in the CNS pain processing network. Hypothetically, a disinhibition of the central endogenous pain modulation may facilitate the development of headache in MS patients.

## Data Availability

On request made available by the corresponding author.

## Abbreviations

MRI: Magnetic resonance imaging
VLSM: Voxel-based lesion symptom mapping
